# Evaluating the planning efficiency for repetitive construction projects using Monte Carlo simulation technique

**DOI:** 10.1038/s41598-025-12779-w

**Published:** 2025-07-28

**Authors:** Ahmed M. Ebid, Taher Ammar, Ibrahim Mahdi, Hosam Hegazy

**Affiliations:** 1 Department of Structural Engineering and Construction Management, Future University in Egypt, New Cairo, Egypt; 2 Nile Engineering Consulting Bureau (NECB), Nasr City, Egypt

**Keywords:** Planning efficiency, Repetitive projects, Highway projects, Monte carlo simulation, Stochastic modeling, Resource allocation optimization, Construction scheduling, Uncertainty management, Civil engineering, Applied mathematics

## Abstract

Efficient planning and scheduling are critical for the success of repetitive construction projects, particularly highway infrastructure, which underpins economic growth in developing regions. Traditional scheduling methods often rely heavily on planner experience, limiting their ability to manage uncertainties and resource fluctuations in large-scale projects. This study proposes a Monte Carlo simulation-based framework to enhance planning efficiency by systematically modeling activity prioritization, resource allocation, and schedule optimization. Eighteen hypothetical project cases were analyzed under varying conditions to capture a wide range of uncertainties. The results demonstrated substantial improvements in project duration and resource utilization efficiency compared to conventional methods. Validation using three real-world highway projects in Egypt confirmed the framework’s practical applicability, achieving efficiency improvements of up to 80%. This research offers a data-driven, adaptable approach to repetitive project planning, providing planners with a robust tool to mitigate uncertainties and optimize project outcomes.

## Introduction and background

Construction is an economic development backbone, and it contributes significantly to infrastructure development and societal well-being. The industry is prone to a number of challenges, however, particularly in planning and scheduling of projects, which are extremely important for timely and successful project delivery. Repetitive construction projects, such as skyscraper buildings, highway constructions, and pipeline constructions, have inherent scheduling issues with them because of their repetitive in nature needing effective coordination of resources, workforces, and activities across multiple units or locations^1^. The COVID-19 pandemic has further exacerbated these challenges, particularly by intensifying the need for sustainable project management practices in infrastructure development^1,2^. As a result, the demand for advanced planning and scheduling techniques that are capable of managing uncertainties and complexities of repetitive construction projects has grown.

The conventional methods, such as the Critical Path Method (CPM), have dominated the construction project management for the majority of the time. The techniques, nevertheless, do not suit repetitive projects because they cannot manage continuity of work and resource circulation among units^3^. Line of Balance (LOB) methodology has been proposed as an alternative to repetitive project scheduling, but like the others, it too is limited, particularly in handling uncertainties and non-linear production rates^4^. Recent research has explored a number of optimization models and algorithms in an attempt to improve repetitive construction project scheduling. For instance, deterministic methods are employed and particle swarm algorithms are created to synchronize crews and minimize project duration in repetitive projects^5^. Similarly, integrated models with emergent algorithms and evolutionary optimization techniques are created to plan non-unit repetitive projects with correlated uncertainties^6^.

Despite these advancements, planning and scheduling of repetitive construction projects continue to be plagued with uncertainties such as delays, resource availability, and unforeseen interruptions. These uncertainties can affect project outcomes to a large extent, leading to cost escalation, delays, and loss of quality^7^. To combat these problems, researchers have increasingly utilized simulation techniques, for instance, Monte Carlo simulation, which has the ability to model uncertainties and approximate their impact on project schedules. Monte Carlo simulation has been widely used in numerous fields, including construction management, for simulating complicated systems and approximating the likelihood of different results under uncertainty^8^.

Monte Carlo simulation in construction project management has been explored in several research works. Tokdemir et al.^8^ introduced a delay risk analysis method for repetitive construction projects using Monte Carlo simulation to quantify the impacts of uncertainties on project schedules. Bakry et al.^7^ proposed an optimum repetitive project scheduling algorithm under uncertainty using fuzzy set theory and Monte Carlo simulation to model uncertainty and optimize the schedule. The experiments prove the applicability of Monte Carlo simulation to construction schedules, particularly that of repetitive ones, growing stronger and more robust.

In addition to Monte Carlo simulation, other advanced techniques were also put forward to make construction project planning and repetitive construction project scheduling stronger. Nguyen et al.^9^ applied a fuzzy logic approach and Symbiotic Organism Search (SOS) algorithm for determining the optimal time-cost-quality trade-off in repetitive projects. Gouda et al.^4^ also proposed a hybrid approach to linear repetitive project scheduling based on graph theory to determine the optimal resource allocation and crew routing. These research efforts demonstrate the potential of combining simulation techniques with optimization algorithms to enhance repetitive project scheduling efficiency and effectiveness.

Simulation and optimization techniques have been combined for other applications including resource leveling and crew scheduling. Dai et al.^10^ introduced a combined approach to resource leveling of repetitive projects, with interruptions and variable resource usage for minimizing resource variability. Hegazy and Kamarah^11^ formulated an optimization scheduling model of schedules for repetitive projects with broken activities based on the movement cost and time of crews between locations and on activity delay effect on continuity of work of crews. These findings refer to the importance of considering resource constraints along with break interferences in repetitive scheduling in projects and in simulation models for representing as well as excluding these limitations.

Also, how the learning curves and productivity gains in repetitive construction operations have been academically researched has been investigated perfectly. Ralli et al.^12^ examined the application of various learning curve models in construction productivity analysis to include learning effects in planning and scheduling of construction projects. Biruk and Rzepecki^13^ have proposed a model of repetitive construction tasks based on the learning-forgetting theory, including the impact of learning and interruptions on crew productivity. The study emphasizes the need to account for learning effects and productivity improvement in repetitive project planning and scheduling and the potential for application of simulation methodology in modeling these effects.

Simulation methodology has also not only been applied in resource scheduling and planning in construction project management. Simulation has been applied in certain aspects of construction management, such as risk analysis, cost estimation, and decision-making. Abdallah and Marzouk^14^ suggested a model for planning tunnel construction projects through computer simulation and fuzzy decision-making that assists contractors in deciding on construction time and cost. Puri and Martinez^15^ presented continuous stochastic modeling of construction operations for discrete event simulation with focus on complexity and potential resolution of simulating complex construction processes. These studies demonstrate the potential of simulation techniques in addressing most of the issues in construction project planning and scheduling, particularly repetitive projects.

Although there is considerable potential in simulation-based approaches for improving planning and scheduling of repetitive construction projects, there are many challenges and limitations that should be addressed. One of the main challenges is how to model uncertainty and its effects on scheduling the project. While Monte Carlo simulation is a good method for simulating uncertainties, it requires good input data and assumptions, which are difficult to obtain in practice^8^. In addition, integrating the simulation techniques with optimization algorithms and other advanced procedures can be computationally intensive, particularly for large projects with numerous activities and resources^6^. The application of simulation techniques in construction project management is limited by the access to standard techniques and tools as well as experience and skills^16^.

In order to overcome the above obstacles, more research and development in the application of simulation techniques to the management of construction projects, and especially repetitive projects, are required. This includes developing more accurate and efficient simulation models, integrating simulation techniques with other advanced techniques, and standardizing simulation tools and techniques. More case studies and applications of simulation techniques on actual construction projects are also required, to demonstrate their usefulness and potential advantages.

Scheduling and planning for repetitive construction projects present distinct complexities, particularly under conditions of uncertainty and resource constraints. Traditional scheduling techniques often fall short in addressing these challenges, highlighting the need for more advanced approaches. Monte Carlo simulation offers a powerful platform for modeling uncertainties and quantitatively assessing their impact on project durations, resource allocation, and crew scheduling. Despite its potential, challenges remain, including the complexity of accurately modeling uncertainty, the high computational demands of simulation techniques, and the absence of standardized industry practices or widely adopted tools. Further research and development are essential to enhance the application of simulation methods in construction project management and to improve the overall effectiveness and efficiency of repetitive project scheduling.

Recent studies have advanced this field by integrating Monte Carlo simulation with other innovative techniques to better manage construction uncertainties^17–21^.

This study aims to bridge these gaps by introducing a novel framework based on Monte Carlo simulation. The proposed methodology integrates dynamic resource allocation, activity prioritization, and stochastic variability in input parameters, providing a robust and practical tool for evaluating and optimizing planning efficiency. By simulating multiple scenarios, this approach offers construction industry stakeholders actionable insights to enhance project scheduling and resource utilization, leading to more predictable and efficient project outcomes.

### Problem statement and research contribution

Planning and scheduling repetitive construction projects, such as highways, involve distinct challenges arising from their large scale, complex activity interdependencies, and inherent exposure to uncertainties. Traditional scheduling techniques, including the CPM and repetitive project-specific methods like the LOB, largely depend on the expertise and intuition of planners. However, these methods often assume constant productivity rates and fixed resource allocations, making them less effective in accommodating real-world variations in resource availability, productivity dynamics, and unexpected project disruptions.

In practice, this results in inefficient resource utilization, increased project durations, and higher costs. Existing optimization approaches have shown potential but are often limited by computational complexity and the requirement for detailed, often unavailable, input data.

This research addresses these gaps by developing a Monte Carlo simulation-based framework tailored for repetitive construction projects. The framework systematically models uncertainty by simulating variability in productivity rates, resource allocations, and activity sequences. Unlike existing approaches, it integrates a spreadsheet-based tool that enables accessible, scalable application in real-world project planning without the need for complex software or extensive computational resources.

The novelty of this study lies in:

- Introducing a stochastic simulation approach to optimize both project duration and resource utilization efficiency under uncertainty.
- Validating the framework through real-world case studies from highway projects in Egypt.
- Providing a practical, easy-to-implement tool for construction planners in resource-constrained environments.

## Methodology

**Fig. 1:**
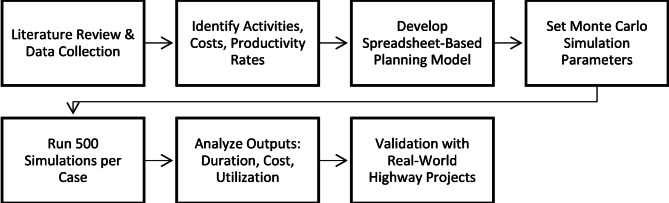
Research methodology.

The study follows a structured methodology, combining data collection, spreadsheet modeling, and Monte Carlo simulation to evaluate and optimize planning efficiency for repetitive highway construction projects. The methodology began with the collection, organization, and analysis of relevant literature to:

- Identify research gaps,
- Determine commonly performed activities in highway projects,
- Establish the precedence and priority relationships among activities,
- Estimate the average unit costs and production rates associated with each activity (Fig. 1).

### Data collection and activity analysis

**Fig. 2:**
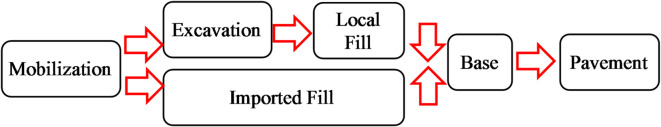
The considered priorities and prerequisites.

A comprehensive review of relevant literature and industry reports was conducted to identify the key activities common to highway construction projects. For each activity, typical unit costs, productivity rates, and precedence relationships were collected and summarized. Table 1 summarizes the main activities of the highway projects and commonly used values for their unit cost and crew productivity, while the generic time schedule shown in Fig. 2 illustrates the priority and prerequisites of each activity. The mobilization duration and cost were considered 10% of the total project duration and cost, respectively.

**Table 1:** The commonly used values for activities unit cost and crew productivity.

Activity	Average unit cost^a^ ($/m^3^)	Crew productivity^b^ (m^3^/day)	Average crew productivity^b^ (m^3^/day)	Crew cost rate^a^ (1000$/day)	Normalized crew cost rate
Excavation	2	400–800	600	0.8–1.6	0.05
Local fill	4	800–1600	1200	3.5–7.0	0.15
Imported fill	8	1200–2400	1800	10.0–20.0	0.50
Base^c^	12	800–1600	1200	10.0–20.0	0.50
Pavement^d^	160	120–240	180	20.0–40.0	1.00

^a^Average rates in 2020, 10% annual rate was considered.

^b^16 working hours per day was considered.

^c^Unified base thickness of 40 cm was considered.

^d^Unified asphalt thickness of 12 cm was considered.

### Development of the planning model

A dynamic spreadsheet-based model was developed to simulate the planning schedule of highway projects based on the collected activities and their priorities and prerequisites. The generated time schedule was used to calculate the total duration, cost, and the cash in distribution with time for the project based on the input duration, number of crews, and cost of crew for each activity.

### Monte Carlo simulation setup

Monte Carlo simulation technique was implemented to conduct a full parametric study using the developed spreadsheet. The study included eighteen cases for the same project size (150 km length, 20 m width and divided into 15 segments 10 km each); each case had a different combination of excavation, fill, base, and pavement quantities. The quantities of the considered eighteen cases are summarized in Table 2.

**Table 2:** The considered quantities for each case of the parametric study.

Case ID	Average Excavation depth (m)	Average local fill height (m)	Average Imported fill height (m)	Base thickness (m)	Pavement thickness (m)
000	0.00	0.00	0.00	0.40	0.12
002	0.00	0.00	2.00		
004	0.00	0.00	4.00		
200	2.00	0.00	0.00		
202	2.00	0.00	2.00		
204	2.00	0.00	4.00		
220	2.00	2.00	0.00		
222	2.00	2.00	2.00		
224	2.00	2.00	4.00		
400	4.00	0.00	0.00		
402	4.00	0.00	2.00		
404	4.00	0.00	4.00		
420	4.00	2.00	0.00		
422	4.00	2.00	2.00		
424	4.00	2.00	4.00		
440	4.00	4.00	0.00		
442	4.00	4.00	2.00		
444	4.00	4.00	4.00		

For each case, Monte Carlo simulation was used to generate 500 random combinations the spreadsheet inputs (productivity per crew, number of crews and normalized cost of crew for each activity) within the following limits:

- Productivity per crew for each activity, the average value from Table 1 ± 50%.
- Number of crews per activity, between (1 and 10).
- Normalized cost of crew for each activity, the value from Table 1 ± 25%.

These limits were chosen based on the collected data from literature review.

### Performance metrics

For each simulation, the developed spreadsheet generated the time schedule and calculated the total duration, total cost and draw the required (cash in – time) diagram. Then the utilization efficiency and duration efficiencies were calculated for each simulation using Eqs. 1, 2 respectively.

1 $${\text{Utilization}}\:{\text{efficiency}} = \frac{{{\text{average}}\:{\text{cash}}\:{\text{in}}\:{\text{for}}\:{\text{the}}\:{\text{considered}}\:{\text{simulation}}}}{{{\text{maximum}}\:{\text{cash}}\:{\text{in}}\:{\text{for}}\:{\text{the}}\:{\text{considered}}\:{\text{simulation}}}}$$

2 $${\text{Duration}}\:{\text{efficiency}} = 1 - \frac{{{\text{total}}\:{\text{duration}}\:{\text{of}}\:{\text{the}}\:{\text{considered}}\:{\text{simulation}}}}{{{\text{max}}.\:{\text{total}}\:{\text{duration}}\:{\text{of}}\:{\text{all}}\:{\text{simulations}}\:{\text{of}}\:{\text{the}}\:{\text{considered}}\:{\text{case}}}}$$

**Fig. 3:**
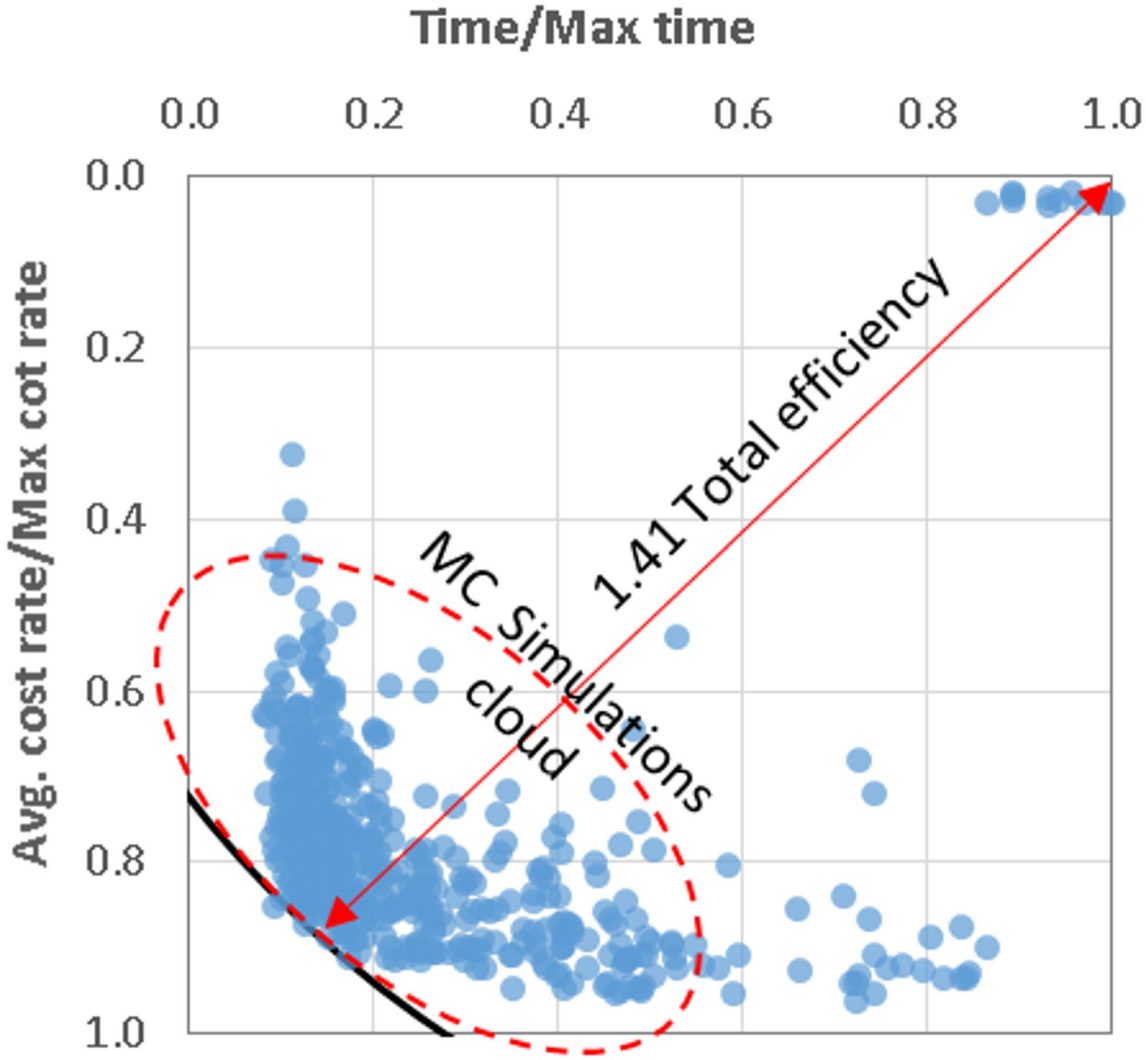
Typical output of the developed spreadsheet showing the Mont Carlo simulation cloud and the total efficiency.

The calculated utilization and duration efficiencies from all simulations were graphically presented as cloud of points as shown in Fig. 3. The total efficiency of any simulation could be calculated using Eq. 3. The optimum simulation was defined as the one that have maximum total efficiency.

3 $${\text{Total}}\:{\text{efficiency}} = \sqrt {\frac{{{\text{Utilization}}\:{\text{efficiency}}^{2} + {\text{Duration}}\:{\text{efficiency}}^{2} }}{2}}$$

#### Validation through real projects

To validate the framework, three real-world highway projects in Egypt were analyzed. Actual project data, including durations, crew sizes, and productivity rates, were compared against simulated results to assess the framework’s predictive accuracy and practical applicability.

## Results

The outcomes of this study were the optimum simulation for each of the eighteen considered cases; this includes the duration, cost per day, and number of crews for each activity besides total duration, total cost, and finally, the efficiencies (utilization, duration, and total). Tables 3 and 4, and 5 summarized the optimum number of crews, optimum normalized cost per day, and optimum duration of each activity for each considered case, respectively. In addition, Table 6 lists the corresponding utilization, duration, and total efficiencies for each case.

**Table 3:** Optimum number of crews of each activity for each case.

Case ID	Mob	Cut	Local fill	Import fill	Base	Pavement
000	1	0	0	0	2	9
002	1	0	0	2	6	10
004	1	0	0	3	8	10
200	1	8	0	0	6	10
202	1	8	0	1	9	9
204	1	6	0	4	6	10
220	1	6	10	0	9	9
222	1	8	9	9	6	10
224	1	7	3	9	1	9
400	1	6	0	0	7	10
402	1	9	0	4	8	10
404	1	9	0	1	2	10
420	1	10	7	0	4	10
422	1	9	2	8	7	10
424	1	9	9	2	9	10
440	1	3	5	0	2	10
442	1	9	6	9	1	8
444	1	6	2	9	2	10

**Table 4:** Optimum normalized cost per day of each activity for each case.

Case ID	Mob	Cut	Local fill	Import fill	Base	Pavement
000	0.22	0	0	0	0.41	1.18
002	0.26	0	0	0.50	0.59	1.22
004	0.25	0	0	0.62	0.49	1.14
200	0.22	0.04	0	0	0.50	1.03
202	0.21	0.05	0	0.39	0.51	0.91
204	0.25	0.06	0	0.61	0.41	1.25
220	0.26	0.06	0.12	0	0.57	1.23
222	0.26	0.05	0.18	0.47	0.59	1.21
224	0.23	0.05	0.14	0.51	0.45	1.07
400	0.24	0.04	0	0	0.57	1.20
402	0.22	0.04	0	0.43	0.41	1.14
404	0.26	0.06	0	0.59	0.60	1.19
420	0.25	0.05	0.12	0	0.59	1.12
422	0.21	0.04	0.16	0.40	0.56	0.88
424	0.23	0.04	0.17	0.48	0.45	1.12
440	0.25	0.05	0.14	0	0.50	1.16
442	0.23	0.05	0.18	0.50	0.53	1.02
444	0.24	0.05	0.16	0.41	0.55	1.17

**Table 5:** Optimum duration (days) of each activity for each case.

Case ID	Mob	Cut	Local fill	Import fill	Base	Pavement
000	2	0	0	0	9	8
002	4	0	0	16	4	13
004	4	0	0	25	5	7
200	8	65	0	0	3	7
202	7	42	0	12	5	10
204	14	92	0	28	5	8
220	15	94	36	0	7	7
222	13	65	30	14	4	10
224	18	96	28	24	10	14
400	11	92	0	0	3	9
402	17	129	0	15	5	12
404	17	119	0	24	6	13
420	22	183	18	0	4	8
422	17	92	36	17	4	12
424	16	85	19	34	5	8
440	14	80	36	0	8	7
442	17	68	60	19	7	11
444	23	107	37	52	7	20

**Table 6:** Corresponding utilization, duration and total efficiencies for each case.

Case ID	Utilization efficiency	Duration efficiency	Total efficiency
000	0.73	0.77	0.75
002	0.78	0.76	0.77
004	0.77	0.87	0.81
200	0.86	0.91	0.88
202	0.86	0.87	0.87
204	0.84	0.83	0.84
220	0.87	0.83	0.85
222	0.82	0.86	0.84
224	0.86	0.80	0.83
400	0.91	0.92	0.92
402	0.88	0.91	0.89
404	0.87	0.87	0.87
420	0.90	0.88	0.89
422	0.88	0.90	0.89
424	0.86	0.91	0.88
440	0.93	0.86	0.89
442	0.85	0.91	0.88
444	0.86	0.87	0.86

## Discussion

**Fig. 4:**
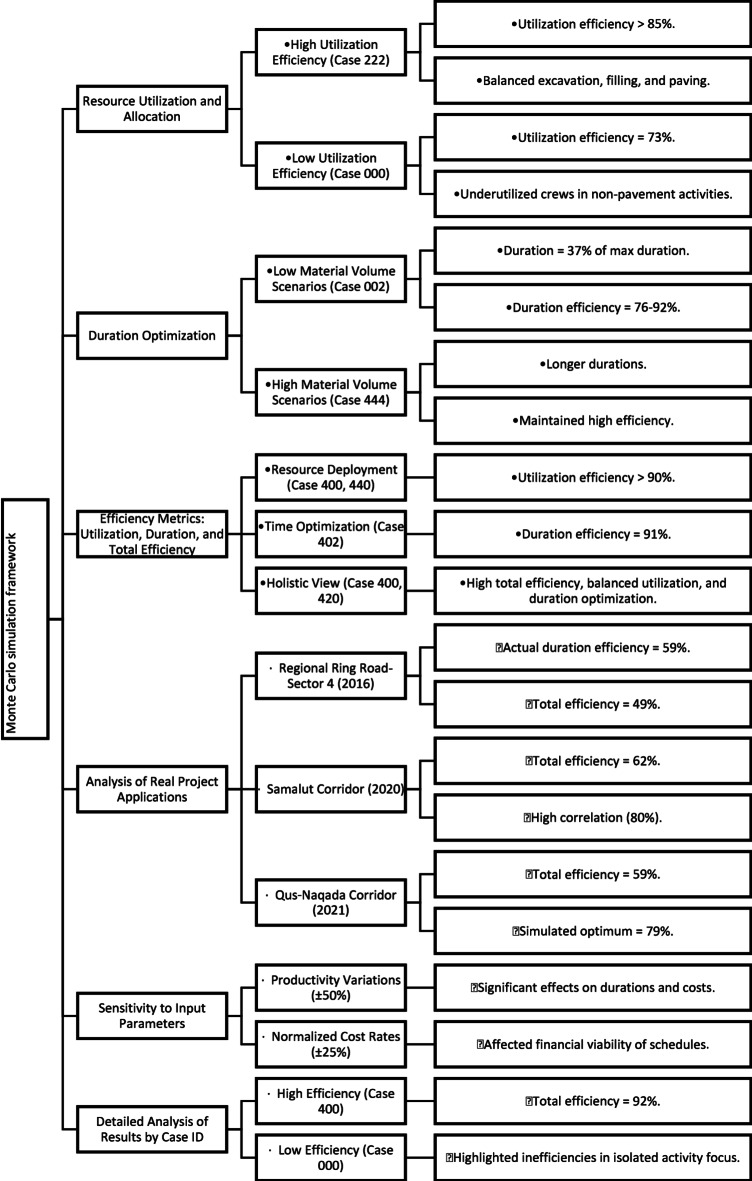
Summary of the applications of the Monte Carlo simulation framework.

**Fig. 5:**
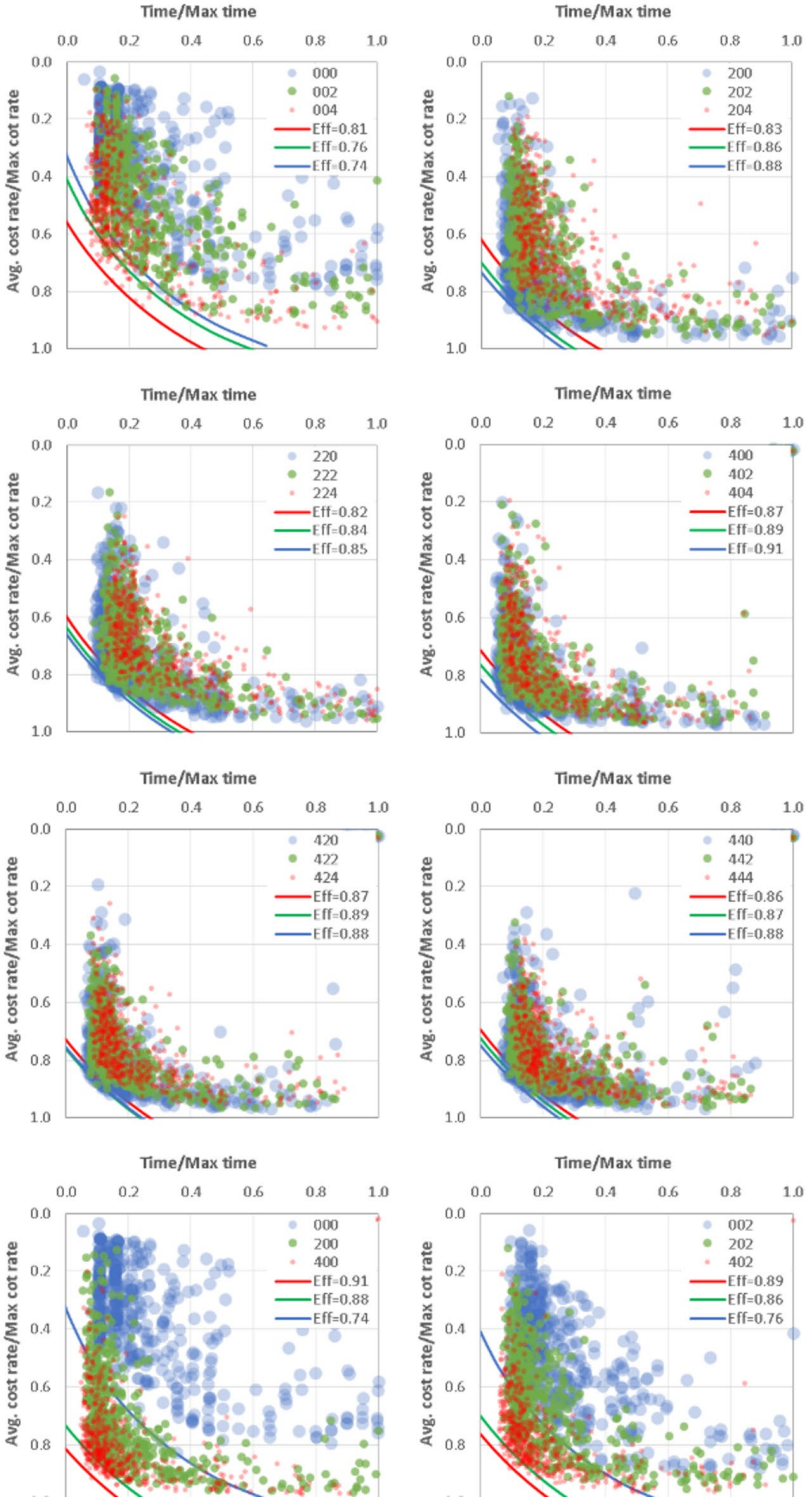
Analysis of cost and time performance in different scenarios.

Due to static allocation practices, traditional methods often lead to inefficient use of resources. In contrast, the Monte Carlo simulation framework enables dynamic adjustments in crew sizes and productivity rates. The simulation results identified optimal crew compositions for each activity, thereby reducing idle times and promoting steady resource utilization throughout project phases. This adaptability minimizes resource wastage and enhances overall efficiency. The results indicate that the Monte Carlo simulation framework can improve planning efficiency in repetitive construction projects. The model identifies optimal schedules and resource allocations by simulating various scenarios with random inputs for productivity rates, crew numbers, and normalized costs, as shown in Figs. 4 and 5.

### Verification

Three real-life highway projects were used to verify the assessment approach that had been developed. All these projects were constructed in Egypt between 2016 and 2021. For each project, both utilization and duration efficiencies were calculated based on the actual quantities, prices, time schedule, number of crews and crew productivity. In addition, the total efficiency was estimated and compared with the closest case total efficacy from Table 6.

#### Case study 1: regional ring road-sector 4 (2016)

The project was a highway segment 1.9 km length and 40 m width in Cairo governorate. The average excavation depth, backfilling height and base layer thickness were 1.0 m, 0.75 m, and 0.4 m respectively. The schedule gave 20, 31, 27, and 58 days for excavation, backfilling, base layer and pavement layer. The actual numbers of crews were 8, 2, 1, and 1 for te previous activities in order. The maximum calculated duration was 172 days, the actual duration was 71 days, and hence, the duration efficiency was 59%, while the utilization efficiency was 36%. Accordingly, the total efficiency was 49%. This case is an average between (000) and (220), hence the optimum total efficiency is between (75 & 85%) ≈70%, which indicates a fair planning (49 / 70 = 70%).

#### Case study 2: Samalut corridor (2020)

This was a 30 km length, 20 m width highway crossing the Nile River from east to west at Samalut city in Minya governorate. The average excavation, embankment, base layer, and pavement layer were 0.10, 2.00, 0.40, and 0.15 m in order. The duration of these activities were 80, 60, 120, and 162 days, respectively. The reported numbers of crews were 1, 10, 2, and 3 for the previous activities in order. Accordingly, the duration efficiency was 77%, the utilization efficiency was 40%, and the actual total efficiency was 62%, while the optimum efficiency for case (002) was 77%. That indicates a very good planning (62 / 77 = 80%).

#### Case study 3: Qus-Naqada corridor (2021)

The last project is another transverse corridor that crosses the Nile river at Qus city in Qena governorate. It is 19 km length and 20 m width. The reported quantities per square meter were 0.15 m, 3.0 m, 0.5 m, and 0.18 m for excavation, backfilling, base, and pavement layers. The planned durations for these activities were 80, 65, 112, and 172 days in order. The numbers of the crews utilized were 1, 10, 2, and 2 for the previous activities, respectively. That makes the maximum duration 1146 days while the planned one was 317 days, hence, both duration and utilization efficiencies were 72% and 41%, respectively. Accordingly, the total efficiency was 59%. Comparing this value with the optimum one (between 002 and 004 ≈ 79%) indicates very good planning (59 / 79 = 75%).

### Sensitivity analysis

To evaluate the robustness of the simulation outcomes, a sensitivity analysis was conducted on key input parameters: productivity rates, number of crews, and crew cost rates. Each parameter was varied within its defined range while holding the others constant to assess its individual impact on project duration and resource utilization efficiency.

The analysis revealed that project duration was most sensitive to variations in productivity rates, with a ± 50% change in productivity resulting in an average ± 20% change in project duration. Variations in the number of crews had a moderate effect, primarily influencing resource utilization patterns, while changes in crew cost rates had a comparatively smaller impact on total efficiency metrics.

**Fig. 6:**
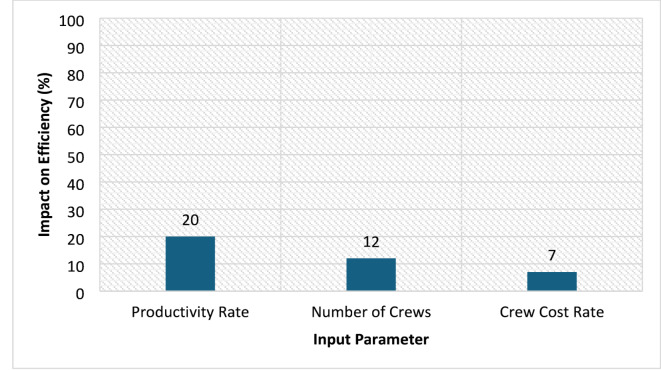
Sensitivity of efficiency metrics to input parameters.

Figure 6 illustrates the relative sensitivity of each parameter to total efficiency. These findings confirm that while all three parameters affect planning efficiency, productivity rate fluctuations play a dominant role in determining project outcomes.

## Conclusions

Efficient planning of repetitive construction projects is essential for delivering infrastructure projects on time and within budget, particularly in developing countries where resource constraints are common. Traditional scheduling methods often fall short in managing uncertainties and dynamic resource allocation, leading to inefficiencies and project delays.

This study introduces a Monte Carlo simulation-based framework that integrates stochastic modeling of productivity rates, crew allocations, and cost parameters within a dynamic, spreadsheet-driven environment. The proposed approach addresses critical gaps in traditional planning by providing a robust tool for optimizing both project duration and resource utilization under uncertainty. Validation through real-world highway projects demonstrated that the framework can achieve planning efficiency improvements of up to 80%. The key contributions of this research are:

- Developing an accessible and practical simulation tool that does not require specialized software.
- Demonstrating the importance of considering stochastic variations in productivity and resource use in repetitive construction planning.
- Providing empirical validation through case studies, enhancing the credibility and applicability of the approach in real-world contexts.

### Limitations and future work

While the framework effectively models uncertainty and optimizes planning efficiency, the current study is limited to highway construction projects in Egypt. Further research is needed to test the framework across different types of repetitive projects, such as pipelines and high-rise buildings, and in varying geographic and economic contexts. Additionally, integrating optimization algorithms with the simulation framework could further enhance decision-making capabilities and efficiency outcomes.

While the proposed framework demonstrated significant improvements in planning efficiency for repetitive construction projects, several limitations should be acknowledged:

- The validation was limited to three highway projects in Egypt. Regional differences in labor productivity, cost structures, and resource availability may affect the generalizability of the findings.
- The study focused exclusively on highway construction projects. The framework’s applicability to other types of repetitive projects, such as pipelines or high-rise buildings, requires further investigation.
- The model relies on assumed ranges for productivity rates and cost parameters based on literature data. Access to more extensive empirical datasets could enhance the accuracy and reliability of the simulations.

Future research should explore the application of the framework to a broader range of project types and geographic regions. Additionally, integrating advanced optimization algorithms, such as genetic algorithms or particle swarm optimization, could further enhance the model’s capability to generate optimal planning solutions under uncertainty.

## Data Availability

All data, models, and code generated or used during the study appear in the submitted article.

## Abbreviations

CPM: Critical path method
LOB: Line of balance
MCS: Monte Carlo simulation
DES: Discrete event simulation
SD: System dynamics

## Symbols

E_d_: Duration efficiency
E_u_: Utilization efficiency
E_t_: Total efficiency
C: Total cost
D: Project duration
R: Resource utilization rate
P: Productivity rate (m^3^/day)
N: Number of crews
C_r_: Crew Cost Rate (USD/day)
